# Policies on doctors’ declaration of interests in medical organisations: a thematic analysis

**DOI:** 10.1177/01410768231181248

**Published:** 2023-06-08

**Authors:** Victoria Tzortziou Brown, Margaret McCartney, Patrycja Talaga, Richard Huxtable, Andrew Papanikitas, Elizabeth David-Barrett

**Affiliations:** 1 Wolfson Institute of Population Health, 4617 Queen Mary University of London, London, E1 4NS, UK; 2 School of Medicine, University of St Andrews, St Andrews, KY16 9TF, UK; 3 Centre for Ethics in Medicine, Population Health Sciences, 152331 Bristol Medical School, Bristol, BS8 2PS, UK; 4 Nuffield Department of Primary Care Health Sciences, University of Oxford, Oxford, OX2 6GG, UK; 5 School of Law, Politics and Sociology, University of Sussex, Brighton, BN1 9QE, UK

**Keywords:** Competing interests (ethics), ethics, health policy, medical careers, professional conduct and regulation

## Abstract

**Objectives:**

There has been growing concern about doctors’ conflicts of interests (COIs) but it is unclear what processes and tools exist to enable the consistent declaration and management of such interests. This study mapped existing policies across a variety of organisations and settings to better understand the degree of variation and identify opportunities for improvement.

**Design:**

Thematic analysis.

**Setting:**

We studied the COI policies of 31 UK and international organisations which set or influence professional standards or engage doctors in healthcare commissioning and provision settings.

**Participants:**

31 UK and international organisations.

**Main outcome measures:**

Organisational policy similarities and differences.

**Results:**

Most policies (29/31) referred to the need for individuals to apply judgement when deciding whether an interest is a conflict, with just over half (18/31) advocating a low threshold. Policies differed on the perception of frequency of COI, the timings of declarations, the type of interests that needed to be declared, and how COI and policy breaches should be managed. Just 14/31 policies stated a duty to report concerns in relation to COI. Only 18/31 policies advised COI would be published, while three stated that any disclosures would remain confidential.

**Conclusions:**

The analysis of organisational policies revealed wide variation in what interests should be declared, when and how. This variation suggests that the current system may not be adequate to maintain a high level of professional integrity in all settings and that there is a need for better standardisation that reduces the risk of errors while addressing the needs of doctors, organisations and the public.

## Introduction

Medical ethics recognise the moral obligation of doctors to act for the benefit of their patients (beneficence).^1^ Conflicts of interests (COIs) arise when doctors’ professional judgement and duties are influenced by secondary interests.^2^ These interests may be financial or non-financial and fuel a moral tension between personal interests and professional responsibilities.^3^ COIs can cause harm, thus violating another ethical principle (non-maleficence), even though the harm may be in the future, subtle or not formally reported.^3^ For example, conflicting interests can lead to bias in the design and reporting of clinical trials, resulting in an overestimation of their benefits and an underestimation of their risks.^4^ Evidence shows that professionals alter their practice when financial COIs are present,^5–8^ often underestimating the influence of industry interests.^8,9^ COIs may also undermine public health evidence and impede the development of health processes and policies,^10^ thereby negatively impacting on healthcare provision, equity, justice and costs.^11^

The identification and disclosure of COIs is the first step in analysing risks to patients^3^ and organisations and is being advocated by medical regulators internationally.^12–14^ Since the 1990s, a significant push has been made for medical organisations to implement COI disclosure policies. However, studies within academic and research settings have shown variability in the COI disclosures and suggested that the different definitions of COIs, as well as the ambiguity of disclosure guidelines, make it difficult for academics to know what they need to disclose.^15,16^ In addition, the degree to which these policies are enforced within academic settings can vary, from voluntary questionnaires to mandated full-disclosure, and the interpretation can also be variable, resulting in disclosure discrepancies.^17,18^ In the UK, the Association of British Pharmaceutical Industries has a voluntary register, but a significant amount of funding remains unreported,^19^ partly because healthcare professionals can decline to have their names listed.^20^ However, even when commanding transparency of financial interests through legislation, such as in the case of the Physician Payments Sunshine Act in the U.S., implementation can be inconsistent, arduous and the data difficult to interpret in the absence of contextual and comparative information.^21^

Inconsistencies in the disclosure and management of doctors’ COIs are likely to exist beyond industry-related financial interests and beyond research and academic settings. With the expansion of flexible working and of opportunities to engage in a variety of roles within provider and commissioner organisations, as well as professional bodies, it is likely that an increasing number of doctors may find declaring their interests and complying with the different organisational policies challenging. In the UK, there is currently a debate on whether there is a need for a central register of doctors’ interests.^22^^,^^23^

This study aimed to map existing processes and tools used for doctors’ declaration of interests across a variety of organisations and settings, to better understand the degree of variation and identify good practice and opportunities for improvement.

## Methodology

Between April 2021 and November 2021, we studied the policies of the five largest UK Royal Medical Colleges, the equivalent Medical Colleges in Australia, New Zealand and Canada, the medical regulators in these countries, the American Academy of Family Physicians, the British Medical Association, eight NHS Trusts and seven Clinical Commissioning Groups (CCGs).

The sample strategy was developed with the intention of producing a purposeful sample including organisations that either set or influence professional standards or engage doctors in work related to healthcare commissioning and provision. During scoping work, it was noted that similar debates around declarations of interest were occurring internationally. The country case studies were selected because of their similar professional training and use of English language. We sought to hold relatively constant the formal institutions so as to provide a relevant base for comparing actual practice in these countries with that in the UK and inform learning with realistic potential for policy transfer.

NHS Trusts and CCGs were chosen based on their size and location, trying to ensure wide geographical coverage across the UK.

We searched for COI policies in the organisations’ web home pages. If this search failed to identify a COI policy, we contacted the organisations directly and invited them to take part in the study and send us a copy of their policy, if there was one. The full list of the 34 included organisations is presented in Table 1.

**Table 1.** Organisations included in the study.

Organisations	
UK professional organisations (including medical colleges and medical regulators)	
1	Royal College of General Practitioners
2	Royal College of Physicians
3	Royal College of Surgeons of England
4	Royal College of Surgeons of Edinburgh
5	Royal College of Anaesthetists
6	General Medical Council
7	British Medical Association
Non-UK professional organisations (including medical colleges and medical regulators)	
1	Royal Australian College of General Practitioners
2	Royal New Zealand College of General Practitioners
3	Royal Australasian College of Physicians
4	Royal Australasian College of Surgeons
5	Australian and New Zealand College of Anaesthetists
6	College of Family Physicians of Canada
7	Royal College of Physicians and Surgeons of Canada
8	American Academy of Family Physicians
9	Australian Medical Council
10	Medical Council of New Zealand
NHS organisations (trusts and commissioning organisations)	
1	Manchester University Foundation Trust (the largest provider of specialised services in the Northwest of England)
2	Homerton NHS Foundation Trust (in Northeast London)
3	Guy’s and St Thomas NHS Foundation Trust (in Southeast London)
4	Belfast Trust (the largest integrated health and social care Trust in N Ireland)
5	NHS Greater Glasgow and Clyde (the largest health board in Scotland)
6	Cardiff and Vale University Health Board (one of the largest NHS organisations in Wales)
7	Cumbria, Northumberland, Tyne and Wear NHS Foundation Mental Health Trust (one of the largest mental health and disability Trusts in England)
8	Greater Manchester Mental Health NHS Foundation Trust (one of the largest specialist mental health Trusts in the UK)
9	Oxford University Hospitals (one of the largest NHS teaching Trusts in the UK)
10	North and West London CCG (largest in London)
11	Kent and Medway CCG (largest in Southeast)
12	Norfolk and Waveney CCG (largest in east of England)
13	NHS Devon CCG (largest in Southwest)
14	NHS Black Country and West Birmingham CCG (largest in the Midlands)
15	NHS Leeds CCG (largest in Northeast and Yorkshire)
16	NHS Cheshire CCG (largest in Northwest)
17	NHS England

## Analysis

Thematic and content analyses were carried out, which helped to identify and code key themes and patterns from the study sample through an inductive method.^24^ A thematic mind map was created to explain how the themes and sub-themes related. This process allowed the categorisation of the data into sections that were then cross-analysed.

Deductive content analysis was then used to analyse the policies and assess the frequency of data occurring in different categories.^25^ This analysis helped us identify similarities and differences in the sample of COI policies examined. Each policy was reviewed independently by two members of the research team.

The data were not attributed to individual organisations and were summarised using descriptive statistics.

## Results

Out of the 34 organisations included in the study, two had no policy on the management of COI and one did not respond to two invitations to take part in the study. Most of the remaining organisations (24/31) published their COI policy online. Two policies focused on educational activities.

The length of the policy documents varied between 2 and 47 pages, with an average of 16 pages.

**Figure 1.**
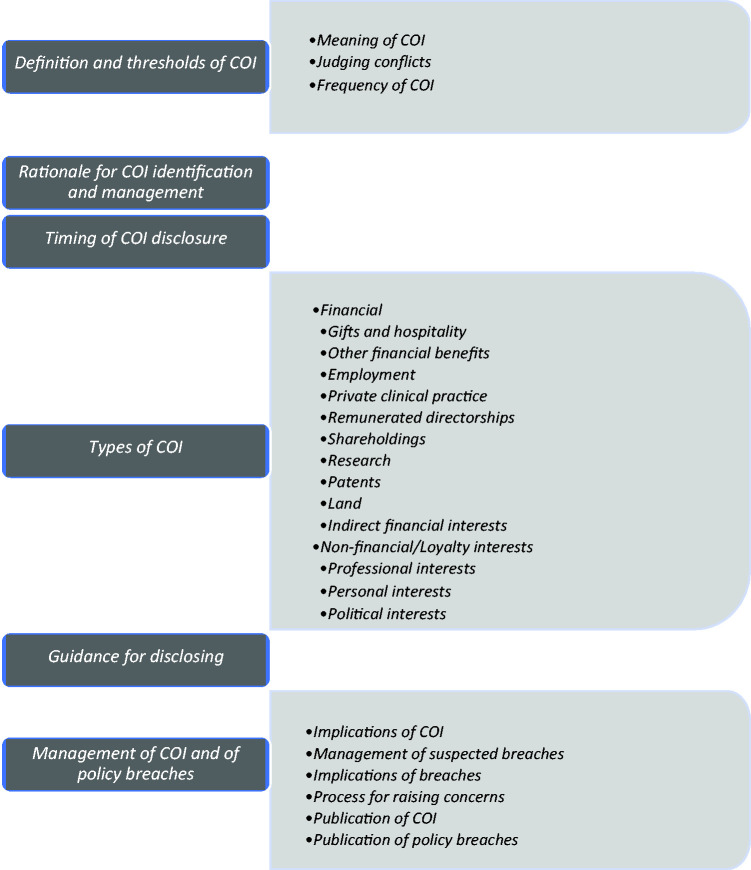
Themes and subthemes of organisational policies on doctors’ declarations of interests. COI: conflict of interest.

The thematic analysis resulted in 6 themes and 24 sub-themes, which presented key concepts identified in the data (Figure 1).

### Theme 1: Definition of COI

One out of the 31 organisational policies did not contain a definition of COI. The definitions in the remaining policies varied and included the collision between different interests – Thompson’s^2^ definition on the potential of interests impairing judgement, and the potential resultant benefit for the individual or third parties.

Most of the policies (29/31) referred to the importance of including actual (‘*where there is a material conflict between one or more interests*’), potential (‘*where there is the possibility of a material conflict between one or more interests in the future*’) and perceived interests (‘*where an observer could reasonably suspect there to be a conflict of interest regardless of whether there is one or not*’).

Eighteen policies (13 from NHS, 2 from UK and 3 from non-UK professional organisations) advocated a low threshold for declaring interests, while three advised that only ‘*relevant*’, ‘*significant*’ *and* ‘*material*’ interests should be declared.

There was a lack of clarity surrounding the scope of interests that physicians were asked to disclose. In certain areas (e.g. research), organisations requested declarations of all interests, while in others (e.g. shareholdings) only conflicts should be declared. Policies also differed on the perception of the frequency of COI. The majority (29/31) of policies referred to the need for applying judgement on which interest can be seen as or create a conflict.

Table 2 gives illustrative quotes on the definition and thresholds of COI.

**Table 2.** Sub-themes and illustrative quotes on the definition and thresholds of conflicts of interest.

Sub-themes	Illustrative quotes
Meaning of COI	'*A conflict of interest will arise where an employee*'*s work-related interests, duties or responsibilities overlap with their private (outside of work) interest, duties, or responsibilities.*’ (Professional organisation)
	‘*The set of conditions in which professional judgement concerning a primary interest tends to be unduly influenced by a secondary interest*.’ (Professional organisation)
	'*Any situation where an individual stands to, or may be perceived to actually or potentially, benefit or alternatively be disadvantaged by a particular decision, either personally or professionally, to the extent it is reasonably possible that the decisions of the person affected may be influenced.*’ (Professional organisation)
Judging COI	'*It is for each individual to exercise their judgement in deciding whether to declare any interests that may be construed as a conflict.*’ (Professional organisation)
	‘*Individuals can have interests without immediately recognising that a potential conflict exists.*’ (NHS organisation)
	'*The test of what constitutes a relevant interest will be whether a reasonable third party would be likely to consider that the objectivity of the person*’*s views or conduct might be affected or influenced by that interest.*’ (Professional organisation)
Frequency of COI	'*Whilst conflicts of interest are rare, it is nevertheless possible that they could happen and could impact adversely on the reputation of the [organisation].*’ (Professional organisation)
	‘*Conflicts of interest are inevitable, but in most cases it is possible to handle them with integrity and probity by ensuring they are identified, declared and managed in an open and transparent way.*’ (NHS organisation)

COI: conflicts of interest; NHS: National Health Service.

### Theme 2: Rationale for COI identification and management

Out of the 31 organisational policies, the majority (26/31) mentioned a general commitment to transparency, integrity or good governance. However, only 18 articulated a clear rationale on why the identification and management of COI needed to take place in a consistent and rigorous way.

The majority of those who provided a clear justification (14/18) were NHS organisations, bound by the relevant legislative requirements and following NHS guidance. A total of 11 NHS policies referred to the Nolan principles, which encompass selflessness, integrity, objectivity, accountability, openness, honesty and leadership.^26^ Out of the 18 policies, 14 (11 NHS, 1 UK professional and 2 non-UK professional), referred to legal requirements due to corporate, charity or NHS-related legislation and 12 NHS policies referred to public financial accountability and that physicians and organisations have a duty to ensure that public money is used not for one’s profit, but for the benefit of the population.

Out of the 15 professional organisations who had a COI policy, only two (one UK and one non-UK) referred explicitly to their duty to promote the declaration and management of COI within their own organisation and set an example reflecting ‘*the highest standards*’.

### Theme 3: Timing of COI disclosure

Table 3 presents the results on the timing of declarations of interests as reported in the 31 organisational policies. In most policies, there was an expectation for updating the declarations at different points during a doctor’s engagement with the organisation.

**Table 3.** Timing of disclosure of declarations of interests.

Timing of disclosure of interest	Number of organisations
Upon appointment	24/31 (15/16 NHS, 6/6 UK professional and 3/9 non-UK professional organisations)
When circumstances change	
Within 28 days	13/31 (10/16 NHS, 2/6 UK professional and 1/9 non-UK professional organisations)
Within 14 days	1/31 (Professional organisation)
As soon as possible	3/31 (1/16 NHS, 2/6 UK professional organisations)
Unspecified	6/31 (2/16 NHS, 2/6 UK professional and 2/9 non-UK professional organisations)
Annually	21/31 (13/16 NHS, 6/6 UK professional and 2/9 non-UK professional organisations)
At each meeting	24/31 (15/16 NHS, 5/6 UK professional and 4/9 non-UK professional organisations)
Prior to elections or when starting new projects	12/31 (9/16 NHS, 2/6 UK professional and 1/9 non-UK professional organisations)
Before educational events	2 Professional organisations whose policy referred to educational activities
Unclear when and how often to declare COI	3/31 (1/16 NHS organisation and 2/9 non-UK professional organisations)

COI: conflict of interest; NHS: National Health Service.

Three policies (two of which were from NHS organisations) specified that retrospective COIs in the previous 12–36 months should also be declared. One policy by a non-UK professional organisation also asked for the declaration of future interests that ‘*are known to be going to occur during the next 12 months*’.

### Theme 4: Types of COIs

Financial interests were identified by all organisations as concerning and, thus, in need of disclosing. Several organisations acknowledged other important categories and sub-categories of financial and non-financial (loyalty) interests, which are presented in Table 4.

**Table 4.** Types of COIs in organisational policies with illustrative quotes.

Type of interest	Number of policies referring to this	Comments and illustrative quotes
Financial	31/31	‘*Where an individual may get direct financial benefit from the consequences of a decision they are involved in making*’ (NHS Organisation)
Gifts and hospitality	22/31 (14/16 NHS, 3/6 UK professional and 5/9 non-UK professional organisations)	There were variations in the amounts of gifts and hospitality that needed to be declared, ranging from £0 to £500. The threshold was set to £50 for the declaration of gifts and £25 for hospitality declarations in 12/16 NHS organisations. Three organisations forbid the acceptance of any gifts or hospitality.
		Policies were often vague and required a degree of judgement with statements such as: ‘*Sponsorship of events by appropriate external bodies will only be approved if a reasonable person would conclude that the event will result in clear benefit for the organisation and the NHS.*’ (NHS organisation)
Other financial benefits	16/31 (8/16 NHS, 4/6 UK professional and 4/9 non-UK professional organisations)	One professional organisation defined ‘benefits’ as ‘*using (organisational) resources for private benefit.*’ Another professional organisation included ‘*Honoraria or fees for speakers or delegates at commercial company organised meetings.*’
		The need for declaring pharmaceutical sponsorship was specifically mentioned in the policies of NHS organisations.
Employment	25/31 (15/16 NHS, 5/6 UK professional and 5/9 non-UK professional organisations)	In 5/31 policies (2 from UK professional and 2 from non-UK professional organisations), outside employment only needed to be declared if the outside role conflicted with the role in the index organisation.
Private clinical practice	14/31 (12/16 NHS, and 2/6 UK professional organisations)	There was variation on the amount of information required when declaring private clinical work. One professional organisation required clinicians to declare ‘*where they practise (name of private facility), what they practise (specialty, major procedures), when they practise (identified sessions/time commitment).*’
Remunerated directorships	20/31 (13/16 NHS, 5/6 UK professional and 2/9 non-UK professional organisations)	There was variation in the type of directorships that needed to be included and whether these were in public or private companies and within or outside the country of interest.
Shareholdings	22/31 (14/16 NHS, 6/6 UK professional and 2/9 non-UK professional organisations)	All policies referring to shareholdings advised the declaration only in situations where shareholdings and ownership interests exist in organisations ‘*which are doing, or might reasonably be expected to do, business with [the organisation]*’. The thresholds for declaring shareholdings varied.
Research	18/31 (14/16 NHS. 2/6 UK professional and 2/9 non-UK professional organisations)	Most of the policies referring to research mentioned that ‘*Funding sources for research purposes must be transparent.*’
Patents	17/31 (12/16 NHS, 3/6 UK professional and 3/9 non-UK professional organisations)	Most NHS organisations required staff to declare patents and other intellectual rights ‘*which are, or might be reasonably expected to be, related to items to be procured or used by the organisation in the course of its normal business activity.*’
Land	4/31 (1/16 NHS, 1/6 UK professional and 2/9 non-UK professional organisations)	The requirements for declaration varied from any commercial holdings, to directly or indirectly leasing, renting, trading, or selling real or personal property to the organisation or use of the organisation’s property for personal advantage.
Indirect financial interests	28/31 (15/16 NHS, 6/6 UK professional and 7/9 non-UK professional organisations)	All policies that referred to indirect financial interests specifically mentioned family members. The majority of these specified the type of family member as those who live within the same household or are a close relative. Twenty-one policies expanded to other close associations such as close friends and associates and business partners.
Non-financial loyalty interests	27/31 (16/16 NHS, 6/6 UK professional and 5/9 non-UK professional organisations)	Most organisations referred to conflicts of loyalty when an individual has a competing obligation or duty to another organisation or person, or religious or political affiliations that could interfere with their ability to make decisions in the best interests of the organisation.
Non-financial professional and personal interests	27/31 (16/16 NHS, 6/6 UK professional and 5/9 non-UK professional organisations)	All NHS and UK professional organisations and 5/9 non-UK professional organisations required the declaration of membership of any voluntary sector board, or lobbying or pressure group with an interest in health and care and possible conflicts due to family relationships.
		Twenty-one policies (from 14 NHS, 4 UK and 3 non-UK professional organisations) referred to the need to declare other personal relationships that could give rise to an actual or perceived conflict of interest. One NHS policy recognised that ‘*these relationships can be hard to define. They are unlikely to be directed by any formal process or managed via any contractual means, however these loyalty interests can influence decision making.*’
Political interests	8/31 (2/16 NHS, 3/6 UK professional and 3/9 non-UK professional organisations)	Policies varied on the requirement to declare political affiliations from: ‘*The [professional organisation] does not want to or need to record information about an individual*’*s political beliefs or views*’ to ‘*Include any party-political involvement by you, your partner or family members.*’ (Professional organisation)
		Most policies advised that political activities can be attended in individual but not organisational representative capacity.

NHS: National Health Service.

### Theme 5: Guidance for disclosing

Most of the policies (28/31) gave examples of potential conflicts to assist understanding. Eighteen policies (nine of which were NHS, five UK and four non-UK professional organisations) had forms for the declaration of interests incorporated into their policy documents and two policies provided links to the forms that were not working. Seventeen of the forms (10 from NHS, 3 from UK and 4 from non-UK organisations) used direct questions, guiding readers on what to include.

 Eleven policies (5 of which were NHS, 3 UK professional and 3 non-UK professional organisations) provided clear details about whom individuals should contact if there was a need for clarification regarding the declarations.

### Theme 6: Management of COI and of policy breaches

Six sub-themes were identified on the management of COI and of policy breaches. The results are summarised in Table 5.

**Table 5.** Management of COI and policy breaches.

Sub-themes	Detailed in the policy	Comments and illustrative quotes
Implications of COI	28/31 (14/16 NHS, 6/6 UK professional and 8/9 non-UK professional organisations)	The implications of COI ranged from just acknowledging the conflict to removing applicable personnel from their role in cases of significant conflict. However, the process of such decision making was often unclear in the policies.
Management of suspected breaches	16/31 (12/16 NHS, 1/6 UK professional and 3/9 non-UK professional organisations)	Nine policies mentioned that breaches would be reported to the audit committee but there were no details on the powers of such a committee to act.
		NHS organisations mentioned their intention to triangulate the declarations with other sources: ‘*Employees should be aware that external organisations, e.g., Association of British Pharmaceutical Industries (ABPI), may also publish information relating to commercial sponsorship or other payments. Such publications will be reviewed to ensure that appropriate internal declarations have been made in accordance with this policy and will take appropriate action where they have not.*’
Implications of breaches	22/31 (14/16 NHS, 3/6 UK professional and 5/9 non-UK professional organisations)	The implications of breaches varied between policies and included disciplinary action that could result in termination of employment, legal action (e.g. in cases of fraud or bribery), withdrawal of professional membership and reporting to the medical regulator.
Process for raising concerns	18/31 (13/16 NHS, 4/6 UK professional and 1/9 non-UK professional organisations)	Fourteen policies (10 from NHS, 2 from UK professional and 2 from non-UK professional organisations) stated that individuals had a duty to report any concerns in relation to COI. One policy stated ‘*Effective management of conflicts of interest requires an environment and culture where individuals feel supported and confident in declaring actual or suspected breaches of the policy.*’ (NHS organisation)
Publication of COI	22/31 (15/16 NHS, 4/6 UK professional and 3/9 non-UK professional organisations)	18/31 policies (15 from NHS and 3 from UK professional organisations) said that the declared interests would be published. One professional organisational policy mentioned that, although the COI would not be published, they would be available to relevant stakeholders on request. Three professional organisational policies explicitly stated that the COI would not be published because they were considered to be confidential, and they would be treated as such.
Publication of policy breaches	13/31 (all of which were NHS organisations)	Seven NHS organisational policies said that anonymised information relating to breaches and how those breaches had been managed would be published on the organisation’s website annually.

COI: conflicts of interest; NHS: National Health Service.

## Discussion

The analysis of organisational policies on the declaration and management of doctors’ COIs revealed wide variation in what should be declared, when and how. There were also variations on how such COIs and policy breaches should be managed and on how transparently they should be communicated.

Our study is limited by the fact that we focused on a sample of policies of professional, regulatory and NHS organisations and we did not investigate the actual processes that institutions use to record and manage COI. However, this is the first study analysing organisational policies on doctors’ COIs, and the findings highlight the degree of variation and the need for standardisation. Similar variations have been found in policies within medical schools and other research institutions.^18^ It is important for both practical and ethical reasons that healthcare professionals know which guidance they should follow and are clear about what they are expected to declare and how to do it.^27^

Only eight policies advised on declaring political activities, and none mentioned religious beliefs. Professional and financial interests can be easier to record, investigate and verify. However, other interests, such as political or religious affiliations, may also cause conflicts,^28^ can be harder to establish and verify and are considered protected characteristics, which may explain their very limited reference in existing COI policies.

The fact that most policies referred to conflicts, rather than interests, presents a challenge for two reasons. First, defining what constitutes a conflict heavily relies on self-perception and individual judgement and is therefore reliant on individual skills to navigate ethical issues and behave impartially. Much of the existing guidance asks the person declaring to consider how much of an interest would constitute a conflict. This can be difficult to gauge – it is often inferred from the amount of a financial stake someone has or from how formally or overtly they have manifested their loyalty to an external cause. Such judgements are made not just on the subject of the declaration but also on whether the interest is current, in the recent or even distant past. In addition, most policies referred to the importance of declaring perceived, and not just actual, conflicts. This is known as ‘the appearance standard’ and is quite common in COI regulation, recognising that the appearance of conflict can in itself undermine trust in the system and therefore cause harm. However, given that existing policies rely to a great extent on self-regulation of COIs, this creates a further layer of complexity: doctors are asked not only to identify and evaluate their own COIs, but also to estimate how these interests will appear to patients and the public.^29^

The second challenge of declaring conflicts rather than interests presents because conflicts are judged in the context of the organisational role or activity the doctor is undertaking. Therefore, the same interest may constitute a conflict for a position in one organisation but not for another. In addition, the very different thresholds for declaring conflicts in existing policies, coupled with the fact that increasingly more doctors are involved in multiple roles in various settings, means that the same doctor may have to submit and update very different COI declaration forms for each of their roles, which is burdensome and increases the risk of mistakes and omissions.

Almost half of the policies lacked a clear rationale for declaring COIs and did not refer to the management of breaches and the process of raising concerns. The policies also differed in their stance on the publication of COIs with conflicting messages on the need for confidentiality versus transparency. There was less variation among NHS organisations, with the majority, albeit not always consistently, following the published guidance on COIs by NHS England. This highlights the positive effect of central standard setting. It has been shown that clear and specific instructions can lead to better compliance and performance.^30^ In addition, policy consistency can assist with the training of doctors and can help with setting clearer expectations for the public. However, the NHS England guidance only applies to NHS Trusts and commissioning organisations^31^ and there is currently no system in place for the declaration of doctors’ interests in other settings such as general practice and primary care.

On the basis of our findings, we propose using the term ‘declaration of interest’ instead of ‘conflict of interest’. This would mean that disclosure is encouraged even of interests that are not necessarily thought by the individual to produce a conflict and would allow independent third-party judgement. Agreed criteria about when a declared interest would be a conflict, and importantly, what action should be taken if so, would assist individuals, organisations and the public with the interpretation of such declarations.

Further research is needed on the expectations of the public on the type of interests that need to be declared, the optimum way of conveying such information and the potential merits, feasibility and acceptability of a well-maintained and protected database of doctors’ interests, with the possibility of both private and public settings, enabling searching and cross-checking through automation. This could work in a similar manner to ORCID, which assists funding bodies and journals to identify authors and applicants. The process should be minimally burdensome for those entering information while being sufficiently comprehensive and reliable for those seeking information,^32^ thereby improving the consistency, efficiency and transparency of declarations of interests.

## Supplemental Material

sj-pdf-1-jrs-10.1177_01410768231181248 - Supplemental material for Policies on doctors’ declaration of interests in medical organisations: a thematic analysis Click here for additional data file. Supplemental material, sj-pdf-1-jrs-10.1177_01410768231181248 for Policies on doctors’ declaration of interests in medical organisations: a thematic analysis by Victoria Tzortziou Brown, Margaret McCartney, Patrycja Talaga, Richard Huxtable, Andrew Papanikitas and Elizabeth David-Barrett in Journal of the Royal Society of Medicine

## Declarations

### Guarantor

VTB.

### Transparency declaration

The lead author (and the article’s guarantor) affirms that the article is an honest, accurate and transparent account of the study being reported; that no important aspects of the study have been omitted; and that any discrepancies from the study as planned (and, if relevant, registered) have been explained.

### Patient and public involvement statement

This study was informed by the Cumberlege Independent Medicines and Medical Devices Safety Review and was inspired by longstanding patient campaigns on the harm from medical interventions and the need for transparent declarations of doctors’ COIs.

### Dissemination to participants and related patient and public communities

We will distribute findings through press releases and on social media, and a plain language summary to relevant patient representative groups. We will share the findings with all participating organisations. We will also disseminate the findings through conference presentations, including invited talks and webinars.

### Data sharing

Reasonable requests for organisational level data should be made to the corresponding author and will be considered. Consent for data sharing was not obtained but most of the policies are publicly available online and the presented data are anonymised.

### Provenance

Not commissioned; externally peer-reviewed.
